# Safety and Efficacy of Nephron‐Sparing Surgery in Bilateral Wilms Tumors: A Systematic Review and Meta‐Analysis

**DOI:** 10.1002/hsr2.71710

**Published:** 2025-12-29

**Authors:** Menna Sarhan, Yasmine Adel Mohammed, Ayah Abdulgadir, Ibrahim Moqbel, Yehia Nabil Abdalla, Salma Allam, Ahmad Alkheder, Khaled Alsayed Abualkhair

**Affiliations:** ^1^ Faculty of Medicine, Zagazig University Zagazig Egypt; ^2^ Department of Pharmacology Faculty of Medicine, Assiut University Assiut Egypt; ^3^ Faculty of Medicine, University of Khartoum Khartoum Sudan; ^4^ Faculty of Medicine, Cairo University Cairo Egypt; ^5^ Faculty of Medicine, Galala University, Galala City Suez Egypt; ^6^ Department of Otorhinolaryngology Al‐Mouwasat University Hospital, Damascus University Damascus Syria; ^7^ Faculty of Medicine, Damascus University Damascus Syria; ^8^ Faculty of Medicine, Syrian Private University Damascus Syria; ^9^ Pediatric Surgery Department Faculty of Medicine, Zagazig University Zagazig Egypt

**Keywords:** bilateral Wilms tumor, meta‐analysis, nephron‐sparing surgery, pediatric oncology, renal preservation, systematic review, Wilms tumor

## Abstract

**Background and Aims:**

Bilateral Wilms tumor (BWT) presents a significant challenge in pediatric oncology, necessitating a delicate balance between achieving oncological control and preserving renal function. While nephron‐sparing surgery (NSS) has emerged as a promising alternative to radical nephrectomy, its overall safety and efficacy profile for BWT remains to be consolidated. This systematic review and meta‐analysis aimed to synthesize the available evidence to evaluate the pooled safety and efficacy outcomes of NSS in children with BWT.

**Methods:**

Following PRISMA guidelines, a systematic search was conducted across five databases (PubMed, Medline, Web of Science, SCOPUS, Cochrane Library). We included studies reporting on pediatric patients with BWT treated with NSS. A single‐arm meta‐analysis was performed using a random‐effects model to pool estimates for survival, recurrence, and renal function outcomes.

**Results:**

Thirteen studies comprising 749 patients were included. The pooled overall survival rate was 55.9% (95% CI: 38.2%–73.7%), relapse‐free survival was 51.4% (95% CI: 32.6%–70.3%), and event‐free survival was 38.0% (95% CI: 23.3%–52.7%). The recurrence rate was 14.9% (95% CI: 9.4%–20.5%). Critically, renal function was well‐preserved, with a mean glomerular filtration rate (GFR) of 92.0 mL/min/1.73 m². The need for dialysis (1.7%) and renal transplantation (4.3%) was low.

**Conclusion:**

NSS provides a viable surgical strategy for BWT, effectively preserving renal function without unduly compromising survival outcomes. The acceptable recurrence and low renal replacement therapy rates underscore its role in mitigating long‐term morbidity. Significant heterogeneity across studies highlights the need for standardized treatment protocols and prospective research to optimize patient selection and outcomes.

**Abbreviations**
BTW: bilateral Wilms tumor
CI: confidence interval
MD: mean difference
NSS: nephron‐sparing surgery
PRISMA: preferred reporting items for systematic reviews and meta‐analyses
RevMan: review manager
WT: Wilms tumor

## 1 Introduction

At around 5% of all pediatric malignancies, Wilms tumor (WT) is the most common kidney cancer in children [1]. WT affects around one out of every 10,000 children, making it a rare yet significant illness [2]. Its significance in pediatric oncology arises not only from its prevalence. but also, from extraordinary advances in treatment regimens that have dramatically improved survival rates over the last several decades [3]. Bilateral Wilms tumor (BWT), which is defined by the presence of tumors in both kidneys at the time of diagnosis, affects 5%–10% of all WT cases. This is a substantial hurdle compared to unilateral WT, with lower overall survival (OS) rates [4]. BWT creates substantially more complex therapeutic issues. Bilateral participation necessitates a careful mix of oncological therapy and renal function maintenance. People are more likely to acquire chronic renal insufficiency. Treatment complexity increases considerably with tumor bilaterality and possible inherited vulnerability [5]. Throughout history, radical nephrectomy was the standard treatment for kidney cancers. While this intensive surgical technique was helpful in preventing the progression of the cancer, it resulted in considerable long‐term renal impairment and related morbidities for the affected children. Loss of renal function can cause a variety of consequences, including the need for dialysis or kidney transplantation, as well as an increased risk of cardiovascular and metabolic illnesses [6]. Nephron‐sparing surgery (NSS) has emerged as a viable alternative for the treatment of BWT in recent years. NSS, also called partial nephrectomy, is designed to remove the tumor while maintaining as much healthy renal tissue as feasible. This strategy has been made possible by advances in neoadjuvant chemotherapy, which can significantly reduce tumor size, boosting the viability of conservative surgical approaches [7, 8, 9, 10, 11]. The primary purpose of NSS is to maintain renal function as much as possible, hence minimizing the long‐term risk of renal failure and its complications. The necessity of retaining the greatest possible long‐term health outcomes in pediatric oncology, as well as the growing emphasis on survivability, supports this. NSS's capacity to retain renal tissue may help children with BWT, reducing the burden of chronic renal disease and its associated morbidities [7, 8, 9, 10, 11]. Despite the potential benefits of NSS, there are ongoing concerns in the literature about its oncologic efficacy, the risk of tumor recurrence, and long‐term results. Furthermore, the heterogeneity in treatment procedures and the lack of established recommendations add to ambiguities in BWT management [12].

This systematic review and meta‐analysis aim to fill this gap by synthesizing the available evidence on the outcomes of nephron sparing surgery in children diagnosed with BWT. Specifically, we aimed to synthesize the available evidence to evaluate the pooled safety and efficacy outcomes—including OS, relapse‐free survival (RFS), event‐free survival (EFS), recurrence rates, and renal function—associated with NSS in children with BWT. The objectives of this review are to evaluate procedural success rates, survival outcomes, and renal function, to identify common complications, and to assess the long‐term outcomes associated with NSS in this unique patient population. By aggregating data from multiple studies, this review seeks to provide a clearer understanding of the efficacy and safety of nephron sparing surgery in children diagnosed with BWTs.

## 2 Methods

### 2.1 Literature Search

Following the PRISMA guidelines and using the PICOS model for inclusion and exclusion criteria [13], we systematically reviewed the literature. We searched five databases [PubMed, Medline, Web of Science Core Collection, SCOPUS, and Cochrane Library].

We included studies that met the following criteria: (1) pediatric patients diagnosed with BWT who were treated with nephron sparing surgery at least in one kidney; (2) Studies are case series, observational studies and clinical trials; (3) Studies published in English. Excluded studies comprised: reviews, case reports, animal studies, conference abstracts, and editorial materials.

While three primary surgical strategies exist for synchronous BWT (bilateral nephrectomy, unilateral NSS with contralateral nephrectomy, and bilateral NSS), the available literature predominantly reports aggregated outcomes for patients undergoing any form of NSS without consistently stratifying results by these specific approaches. Consequently, this review is designed to assess the overall performance of nephron‐sparing techniques where applied, rather than to perform a direct comparative meta‐analysis of the three distinct strategies, for which sufficient discrete data were not available.

Our search terms across databases were: (“wilms tumor” OR “bilateral wilms tumor” OR “nephroblastoma” OR “renal blastoma”) AND (“nephron sparing surgery” OR “partial nephrectomy” OR “kidney sparing surgery” OR “nephron preserving surgery” OR “renal preserving surgery” OR “conservative renal surgery”).

**Figure 1:**
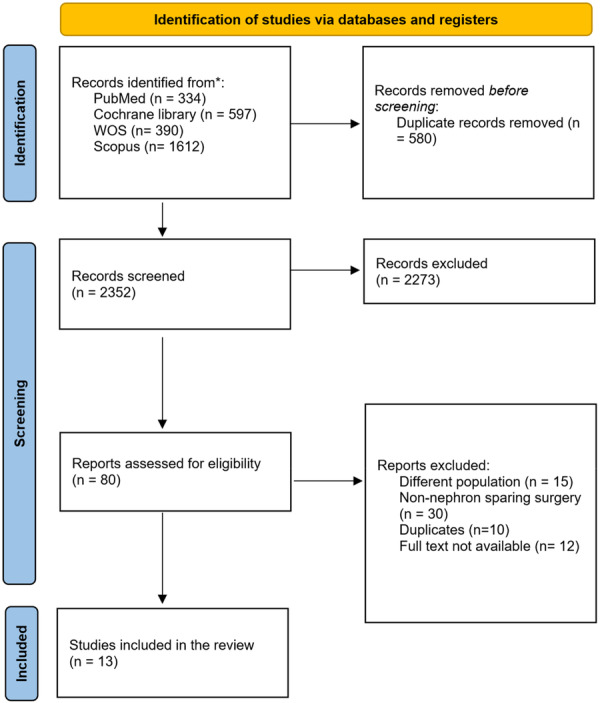
PRISMA flowchart of the study selection process.

Two reviewers independently screened studies based on titles and abstracts, with disagreements resolved by a third reviewer. Full‐text screening against the criteria was conducted using Rayyan software [14]. Detailed screening procedures are presented in the PRISMA flowchart in Figure 1.

### 2.2 Data Extraction Strategy

Two independent reviewers examined databases, excluding studies based on predetermined eligibility criteria from titles and abstracts. They collaboratively assessed each paper's relevance to our review and resolved conflicts or disagreements through team discussions.

The primary outcomes will focus on key indicators, including OS, RFS, and EFS. Secondary outcomes include a recurrence, glomerular filtration rate (GFR), and renal transplant need after surgery.

### 2.3 Risk of Bias Assessment

Two researchers evaluated the quality of the studies included in the analysis by employing the Risk‐of‐Bias in Non‐randomized Studies of Intervention (ROBINS‐I) [15] for non‐randomized studies and NIH tool [16] for observational studies.

### 2.4 Statistical Analysis

All statistical analyses were performed using OpenMeta Analyst software (version 10.14). We conducted a single‐arm meta‐analysis to pool proportions and means along with their 95% confidence intervals (CIs) using a random‐effects model due to anticipated clinical and methodological heterogeneity across studies. Heterogeneity was assessed using the Cochran's *Q*‐test and the *I*² statistic, with significance set a priori at *p* < 0.10 and *I*² > 50% indicating substantial heterogeneity. A leave‐one‐out sensitivity analysis was performed to explore sources of heterogeneity when possible. All hypothesis tests were two‐sided, and a *p*‐value < 0.05 was considered statistically significant. Subgroup analysis was not feasible due to the limited number of studies for each outcome.

## 3 Results

### 3.1 Literature Search

A total of 2933 records were identified from the literature search; 749 were duplicates and removed. Another 2342 records were excluded after the screening process, and only 13 studies were eligible to be included in our meta‐analysis (Figure 1).

### 3.2 Characteristics of the Included Studies

We included 13 studies [5, 6, 8, 9, 10, 11, 17, 18, 19, 20, 21, 22, 23] compromising 749 patients with follow‐up duration ranging from 3.5 [19] to 15 years [9]. The variability in follow‐up periods precluded a standardized time‐point analysis for survival outcomes (OS, RFS, EFS). Only one study was a non‐randomized clinical trial (non‐RCT) [17], and the rest were observational studies [5, 6, 8, 9, 10, 11, 18, 19, 20, 21, 22, 23]. Most children were females. A total of 25.1% of the children had a tumor of Stage I, 16.6% were of Stage II, 23.2% Stage III, 7.5% Stage IV, and 1.9% had Stage V. Histopathology was reported in five studies [9, 17, 18, 20]; most specimens had favorable results, and nearly all the reported metastases were found in the lung. The summary and baseline characteristics of the included studies are reported in Tables 1 and 2.

**Table 1:** Summary of the included studies.

Study ID	Country	Study design	Number	Follow‐up duration, years	Intervention
Agarwala et al. 2014	India	Case series	11	5	Neoadjuvant chemotherapy, surgery, and adjuvant chemotherapy ± radiotherapy.
Alfer et al. 1993	Brazil	Non‐randomized clinical trial	14	4.5	Chemotherapy, radiotherapy, and surgery.
Chen et al. 2023	USA	Retrospective cohort	4	5.6	Chemotherapy and surgery.
Cooper et al. 2000	USA	Retrospective cohort	23	N/A	Chemotherapy and surgery.
Dukes et al. 1992	UK	Case series	10	3.5	Combination of chemotherapy, surgery, and radiotherapy if needed.
Elashry et al. 2012	Egypt	Retrospective cohort	19	5	Chemotherapy followed by nephron‐sparing surgery.
Fang et al. 2023	China	Retrospective cohort	70	6	Nephron‐sparing surgery with preoperative chemotherapy.
Imam et al. 2023	USA	Retrospective cohort	25	NA	Nephron‐sparing surgery or radical nephrectomy.
Kubiak et al. 2004	UK	Case series	23	11	Renal salvage procedures combined with chemotherapy.
Millar et al. 2011	South Africa	Case series	23	15	Nephron‐sparing surgery combined with chemotherapy.
Scalabre et al. 2016	France	Case series	11	11	Nephron‐sparing surgery and total nephrectomy according to SIOP protocols
Sudour‐Bonnange et al. 2024	France, The Netherlands, Spain, Poland, France, Germany, UK, Qatar, Brazil, Switzerland	Prospective cohort	327	7.6	Vincristine and Actinomycin D (AV) chemotherapy regimen, response‐adapted intensification with Doxorubicin if needed
Ehrlich et al. 2017	USA	Prospective cohort	189	3.75	Three‐drug pre‐operative chemotherapy (vincristine, dactinomycin, and doxorubicin) followed by surgery and histology‐based post‐operative therapy

**Table 2:** Baseline characteristics of the included studies.

Study ID	*N*	Age (M ± SD), months	Male [*N* (%)]	Type of nephron surgery	Tumor characteristics, *N* (%)										Presentation [*N* (%)]						
					Histopathology		Tumor stage					Synchronous	Metachronous	Metastasis							
					Favorable	Unfavorable (anaplastic)	Stage I	Stage II	Stage III	Stage IV	Stage V				Mass abdomen	Hematuria	Fever	Pain	Respiratory distress	Congenital anomalies	Predisposing syndromes
Agarwala et al. 2014	11	9 ± 8.6	8 (72.2%)	Tumorectomy: 3 (27.3%) Partial nephrectomy: 6 (54.5%) Nephrectomies (including one nephrectomy done elsewhere): 2 (18.2%)	11 (100%)	0 (0)	N/A	N/A	3 (30%)	N/A	N/A	11 (100%)	0 (0)	Lungs: 2 (18%)	9 (80%)	2 (18%)	2 (18%)	N/A	1 (9%)	1 (9%)	N/A
Alfer et al. 1993	14	NA	2 (14.2%)	Enucleation: 5 (29.4%) Partial nephrectomy: 7 (41.2%) Unilateral Nephrectomy: 5 (29.4%)	4 (28.6%)	1 (7.14%)	0 (0)	10 (71.4%)	4 (28.5%)	0 (0)	0 (0)	14 (100%)	0 (0)	N/A	N/A	N/A	N/A	N/A	N/A	1 (7.1%)	N/A
Chen et al. 2023	4	NA	NA	Partial nephrectomy: 4 (100%)	N/A	N/A	N/A	N/A	N/A	N/A	N/A	N/A	N/A	N/A	N/A	N/A	N/A		N/A	N/A	N/A
Cooper et al. 2000	23	NA	8 (34.7%)	NA	N/A	N/A	9 (39.13%)	4 (17.39%)	7 (30.43%)	2 (8.7%)	N/A	21 (91.3%)	2 (8.7%)	N/A	N/A	N/A	N/A	N/A	N/A	N/A	2 (8.7%)
Dukes et al. 1992	10	NA	NA	Partial nephrectomy: 10 (100%)	10 (100%)	0 (0)	N/A	N/A	N/A	N/A	10 (100%)	N/A	N/A	Lung: 4 (40%)	N/A	N/A	N/A	N/A	N/A	N/A	N/A
Elashry et al. 2012	19	34.6 ± 23.1	6 (31.6%)	NA	12 (63.2%)	7 (36.8%)	7 (36.8%)	9 (47.4%)	2 (10.5%)	1 (5.3%)	0 (0)	19 (100%)		Lung: 4 (21.1%)	N/A	N/A	N/A		N/A	N/A	NA
Fang et al. 2023	70	15.191 ± 26	28 (40%)	NA	NA	NA	26 (37.1%)	25 (35.7%)	14 (20%)	5 (7.1%)	NA	66 (94.3%)	4 (5.7%)	Lung: 5 (7.1%)	49 (70%)	9 (12.9%)	N/A	2 (2.9%)	NA	22 (31.4%)	NA
Imam et al. 2023	25	38.57 ± 45.684	7 (28%)	NA	NA	NA	NA	NA	NA	NA	NA	NA	NA	NA	NA	NA	N/A	NA	NA	NA	NA
Kubiak et al. 2004	23	29.7 ± 47.4	7 (30.4%)	Partial nephrectomy: 18 (39.3%) Tumor excisions: 21 (45.7%) Enucleations: 5 (10.9%) Bench surgical techniques with subsequent autotransplantation: 2 (4.3%)	NA	NA	NA	NA	NA	NA	NA	NA	NA	NA	19 (82.6%)	6 (26%)	5 (21.7%)	4 (17.4%)	NA	NA	NA
Millar et al. 2011	23	NA	8 (34.8%)	NA	16 (69.6%)	2 (8.7%)	4 (17.4%)	14 (60.9%)	1 (4.3%)	0 (0)	4 (17.4%)	18 (78.3%)	5 (21.7%)	Lung: 2 (8.69%), local extra‐renal spread: 2 (8.69%)	18 (78.3%)	NA	NA	NA	NA	NA	NA
Scalabre et al. 2016	11	NA	5 (45.5%)	NA	NA	NA	10 (90.9%)	0 (0)	1 (9.1%)	0 (0)	0 (0)	10 (90.9%)	1 (9.1%)	NA	NA	NA	NA	NA	NA	NA	BWS: 10 (90.9%), Isolated HH: 1 (9.1%)
Sudour‐Bonnange etal. 2024	327	53.9 ± 99.7	85 (49%)	Bilateral nephron sparing surgery (NSS): 48 (28.4%) Unilateral NSS + Contralateral Total nephrectomy (TN): 101 (59.7%) Bilateral TN: 20 (11.8%) Bilateral NSS: 29 (38.1%) Unilateral NSS + Contralateral TN: 40 (52.6%) NSS: 11 (28.5%) Chemotherapy only: 24 (62%)	NA	NA	61 (41.5%)	35 (23.8%)	51 (34.7%)	21 (12%)	NA	NA	NA	34 (20%)	NA	NA	NA	NA	NA	NA	NA
Ehrlich et al. 2017	189	NA	75 (39.8%)	NA	NA	NA	71 (37.50%)	27 (14.40%)	91 (48.10%)	27 (14.40%)	NA	NA	NA	27 (14.4%)	NA	NA	NA	NA	NA	NA	40 (21.1%)

Abbreviations: BWS, Beckwith‐Wiedemann syndrome; HH, hemihypertrophy; M ± SD, mean ± standard deviation; N, number; N (%), number (percent); N/A, not available.

### 3.3 Quality Assessment

**Figure 2:**
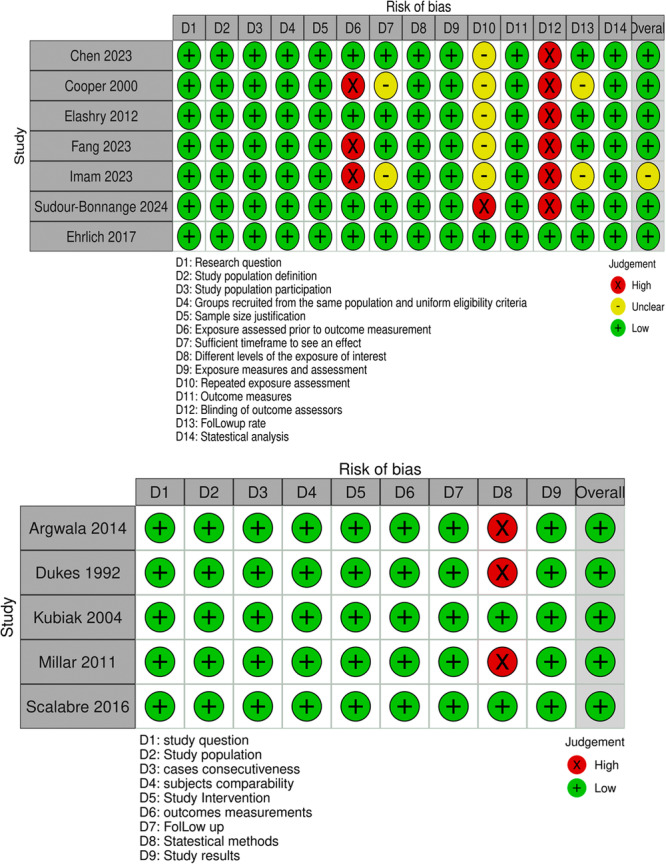
Risk of bias of the included studies.

We used ROBINS‐I to assess the risk of bias in the non‐RCT and NIH tool for all other studies [5, 6, 8, 9, 10, 11, 18, 19, 20, 21, 22, 23]. As shown in Figure 2.

## 4 Outcomes

### 4.1 Chemotherapy

#### 4.1.1 Preoperative Chemotherapy

**Figure 3:**
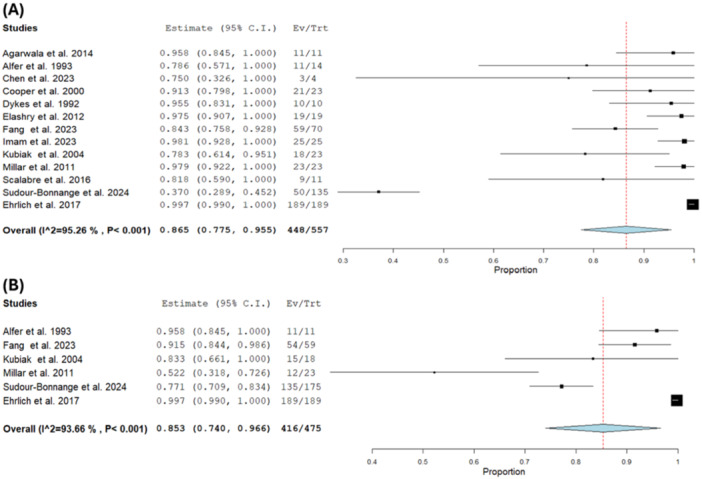
Preoperative chemotherapy. (A) Number of patients taking chemo. (B) Overall response.

We performed a single‐arm meta‐analysis on the rate of preoperative chemotherapy and patient response rate. The pooled estimate of patients with preoperative chemotherapy was 0.865 (95% CI [0.775, 0.955]) with an overall response rate of 0.853 (95% CI [0.740, 0.966]) [5, 6, 8, 9, 10, 11, 17, 18, 19, 20, 21, 22, 23] (*n* = 749). The pooled results showed substantial heterogeneity (*I*² = 95.26%, *p* < 0.001) and (*p* < 0.001, *I* ^2^ = 93.66%), respectively and this heterogeneity could not be solved by sensitivity analysis (Figure 3A,B).

#### 4.1.2 Postoperative Chemotherapy

**Figure 4:**
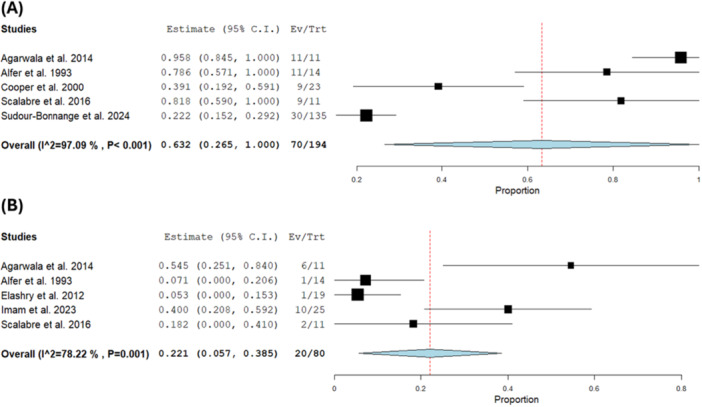
(A) Postoperative chemotherapy. (B) Postoperative radiotherapy after performing leave one out sensitivity analysis.

The overall rate of postoperative chemotherapy was 0.632 with a 95% CI of [0.265, 1.0] [6, 8, 17, 18, 22, 23] (*n* = 575). The result showed substantial heterogeneity (*I*² = 97.09%, *p* < 0.001) that sensitivity analysis could not solve (Figure 4A).

### 4.2 Postoperative Radiotherapy

Five studies [8, 10, 17, 18, 20] (*n* = 80) reported postoperative chemotherapy. The pooled estimate was 0.221 with a 95% CI of [0.0.57, 0.385] and the result was heterogeneous (*I*² = 78.22%, *p* = 0.001), which could not be solved by leaving one out sensitivity analysis (Figure 4B).

### 4.3 Survival Analysis

**Figure 5:**
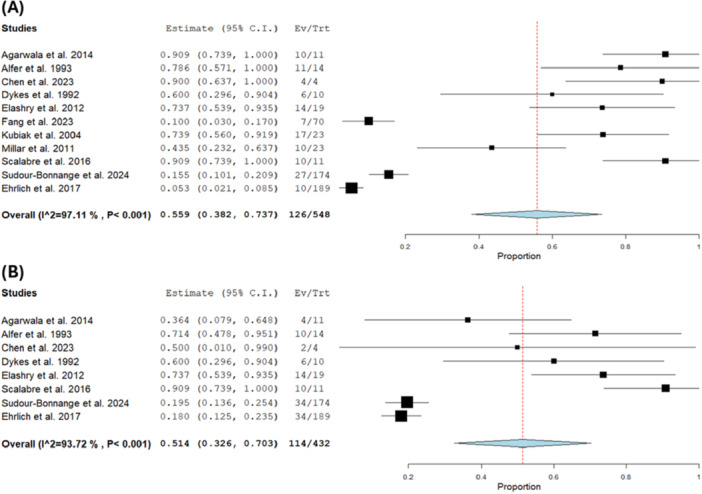
(A) Overall survival. (B) Relapse‐free survival.

The pooled estimate of the OS was 0.559 with a 95% CI of [0.382, 0.737] [5, 8, 9, 11, 17, 18, 19, 20, 21] (*n* = 185) and the pooled estimate showed substantial heterogeneity (*I*² = 97.11%, *p* < 0.001). We could not solve the heterogeneity by leaving one out sensitivity analysis (Figure 5A).

The overall incidence of RFS was 0.514 with a 95% CI of [0.326, 0.703] [5, 8, 17, 18, 19, 20] (*n* = 69). The result was heterogeneous (*I*² = 93.72%, *p* < 0.001), and this heterogeneity could not be resolved by sensitivity analysis, which yielded a non‐significant *Q*‐test (*I*² = 24.04%, *p* = 0.26) (Figure 5B).

**Figure 6:**
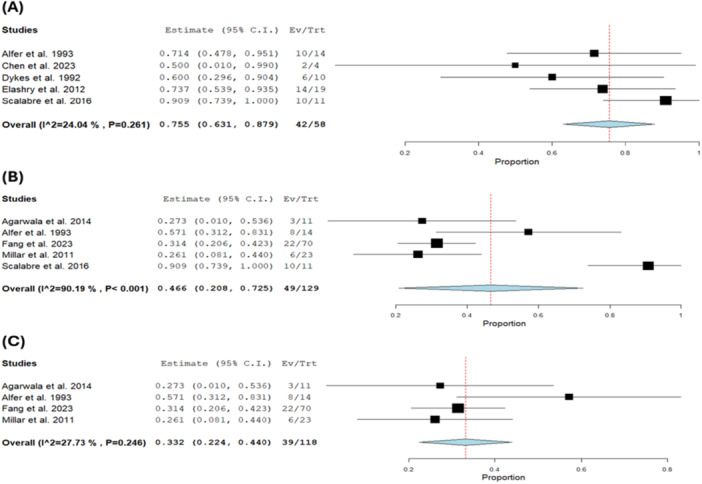
(A, B) Event‐free survival. (C) GFR.

The pooled estimate of EFS was 0.380 with a 95% CI of [0.233, 0.527] [8, 9, 11, 17, 18, 22, 23] (*n* = 645). The result was heterogeneous (*I*² = 91.57%, *p* < 0.001). Heterogeneity was moderately reduced upon exclusion of Scalabre et al. [8] (*I*² = 60.08%, *p* = 0.03) (Figure 6A,B).

### 4.4 GFR

Only two studies [9, 21] (*n* = 46) reported the GFR. These measurements were obtained during long‐term follow‐up (11 years in Kubiak et al. [21] and 15 years in Millar et al. [9]), indicating sustained renal function preservation. The pooled mean was 91.976 mL/min/1.73 m² with a 95% CI of [58.221, 125.732] and the result showed moderate heterogeneity (*I*² = 51.49%, *p* = 0.15) (Figure 6C).

### 4.5 Recurrence

**Figure 7:**
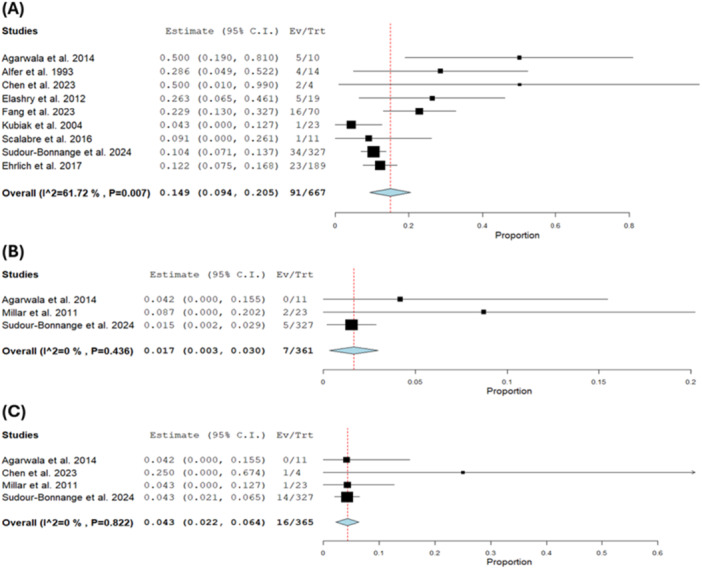
(A) Recurrence. (B) Need for dialysis. (C) Need for renal transplant.

The overall recurrence rate was 0.149 with a 95% CI of [0.094, 0.205] [5, 8, 11, 17, 18, 20, 21] (*n* = 152). The result was heterogeneous (*I*² = 61.72%, *p* = 0.005) and it could not be solved by sensitivity analysis (Figure 7A).

### 4.6 The Need for Dialysis

The need for postoperative dialysis at any point during follow‐up was reported in three studies [18, 21, 22] (*n* = 361). The pooled analysis was 0.017 with a 95% CI of [0.003, 0.030]. The result was homogeneous (*I*² = 0%, *p* = 0.44) (Figure 7B).

### 4.7 The Need for Renal Transplant

The need for a renal transplant was reported in four studies [5, 18, 21, 22] (*n* = 365). The pooled analysis was 0.043 (4.3%) with a 95% CI of [0.022, 0.064]. The result was homogeneous (*I*² = 0%, *p* = 0.82) (Figure 7C).

## 5 Discussion

This systematic review and meta‐analysis of 13 studies, involving 749 patients, provides a comprehensive evaluation of the safety and efficacy of NSS in children with BWTs [5, 6, 8, 9, 10, 11, 17, 18, 19, 20, 21, 22, 23]. The findings suggest that NSS is a feasible approach, achieving a reasonable balance between oncological control and preservation of renal function.

The meta‐analysis revealed an OS rate of 55.9%, a RFS rate of 51.4%, and an EFS rate of 38.0%. These survival rates are notably lower than those reported for unilateral WT, where OS typically exceeds 90% [24, 25]. This discrepancy is expected, as BWT presents a more complex clinical challenge due to bilateral tumor involvement, potentially higher tumor stages, and the necessity to preserve renal tissue, which may limit the aggressiveness of surgical resection [5]. The lower survival rates align with the inherent difficulties of managing BWT, as noted in previous literature [6, 9]. The high heterogeneity (*I*² > 90%) observed in these survival estimates likely stems from differences in patient demographics, tumor stages, follow‐up durations (3.5–15 years), and treatment protocols across studies. For instance, Millar et al. [9] reported outcomes over a 15‐year follow‐up, which may capture more late events compared to shorter‐term studies.

A comparison of outcomes between patients undergoing bilateral NSS versus those treated with unilateral NSS and contralateral radical nephrectomy was not feasible in our meta‐analysis due to the aggregated reporting of outcomes in the primary studies. This represents a critical gap in the literature. Future studies with stratified data reporting are essential to determine if one strategy offers a superior balance of oncological and functional outcomes.

The pooled recurrence rate was 14.9%, a figure that falls within the range observed for unilateral WT [25]. In BWT, however, recurrence poses a unique challenge due to the bilateral nature of the disease and the priority of renal preservation. The use of preoperative chemotherapy in 86.5% of patients, with an 85.3% response rate, reflects its critical role in shrinking tumors to enable NSS [7, 8, 9, 10, 11]. Postoperative chemotherapy was used in 63.2% of cases and radiotherapy in 22.1%, indicating that adjuvant therapies remain integral to achieving oncological control. The heterogeneity in these estimates (*I*² > 78%) suggests variability in their application, possibly influenced by tumor characteristics, institutional protocols, or surgeon preference.

Preserving renal function is a primary goal of NSS. The pooled GFR was 91.98 mL/min/1.73 m² (95% CI: 58.22–125.73); while this value falls within the normal pediatric range and is an encouraging indicator of preserved renal function, this finding is based on only two studies. Therefore, the long‐term durability of this functional preservation requires further validation in larger, prospective cohorts with extended follow‐up [9, 21]. This is further supported by the low rates of dialysis (1.7%) and renal transplantation (4.3%), which contrast favorably with outcomes following radical nephrectomy, where renal insufficiency is more common [6]. The low pooled rate of dialysis (1.7%) is a strong indicator of successful renal preservation, though the aggregated data preclude a distinction between acute post‐operative dialysis and long‐term chronic dialysis. Cooper et al. [6] highlighted the renal‐sparing benefits of conservative surgery in BWT, a finding corroborated by our analysis. The moderate heterogeneity in GFR estimates (*I*² = 51.49%) may reflect differences in measurement methods or follow‐up timing. Nonetheless, the low need for dialysis and transplantation underscores a significant clinical advantage of NSS.

NSS offers a viable approach to managing BWT, achieving acceptable oncological outcomes while significantly preserving renal function. The low rates of dialysis and renal transplantation highlight its advantage in reducing the long‐term burden of renal morbidity. However, the variability in treatment approaches and outcomes emphasizes the need for standardized guidelines and further research to enhance the management of this complex condition.

The absence of randomized controlled trials in BWT management highlights the critical role of large‐scale observational studies and meta‐analyses in synthesizing available evidence. Our study, despite its inherent limitations due to study design heterogeneity, provides valuable pooled outcomes that can inform clinical decision‐making in the absence of higher‐level evidence.

In comparison to the systematic review by Khondker et al. [26], which focused on late renal outcomes following NSS versus radical nephrectomy across both unilateral and BWTs, our findings are largely concordant. Khondker et al. reported significantly better preserved renal function and lower odds of hypertension in patients undergoing NSS, which is consistent with our low rates of dialysis (1.7%) and renal transplantation (4.3%), as well as a mean GFR within the normal pediatric range. However, while Khondker et al. emphasized functional outcomes, our analysis provides additional granularity regarding survival and recurrence metrics specific to BWT—a population with distinctly challenging oncological and functional trade‐offs. This complementary evidence reinforces the dual benefit of NSS: acceptable survival with preserved renal function.

### 5.1 Clinical Implications and Future Directions

The findings affirm that NSS is a practical and effective strategy for managing BWT, offering acceptable survival rates while prioritizing renal preservation. Compared to historical reliance on radical nephrectomy, NSS reduces the long‐term risk of renal failure, aligning with the growing emphasis on survivorship in pediatric oncology [6, 9]. However, the lower survival rates compared to unilateral WT suggest a potential trade‐off between oncological efficacy and renal conservation.

The high heterogeneity across outcomes points to a lack of standardized treatment protocols for BWT. Current practices vary widely, with decisions often guided by tumor size, response to chemotherapy, and institutional experience rather than uniform guidelines. Developing evidence‐based recommendations could enhance consistency and improve outcomes. For example, Fang et al. [11] advocate for refining NSS techniques to optimize its application in BWT, a direction that merits further exploration.

Prospective, multicenter studies—ideally randomized controlled trials—are needed to rigorously assess the efficacy and safety of NSS in BWT. Such studies should incorporate standardized protocols to reduce heterogeneity and provide clearer guidance for clinical practice. Long‐term follow‐up is critical to evaluate not only survival and recurrence but also renal function and late effects. Advances in molecular profiling or imaging, as suggested by Imam et al. [10], could further refine patient selection by identifying those most likely to benefit from NSS. Collaborative efforts, such as those by the SIOP Renal Tumour Study Group, may also contribute valuable insights into optimizing BWT management [22].

### 5.2 Limitations

Several limitations temper the conclusions of this review. The reliance on observational studies, with only one non‐randomized clinical trial, introduces a higher risk of bias compared to randomized controlled trials [17]. The significant heterogeneity in most outcomes limits the generalizability of the pooled estimates, reflecting variability in study designs, patient cohorts, and follow‐up periods. Additionally, the small number of studies reporting certain outcomes, such as GFR, restricts the robustness of these findings. Finally, the absence of long‐term data in some studies may underestimate late events, such as recurrence or renal decline, necessitating cautious interpretation. Furthermore, the most significant limitation was the inability to compare the different surgical strategies (bilateral NSS vs. unilateral NSS with contralateral nephrectomy) due to a lack of discretely reported outcomes in the available literature. This precluded a direct comparison of their long‐term results, which should be a primary focus of future, prospectively designed studies.

## 6 Conclusion

NSS for BWT appears to offer a balanced approach between oncologic control and renal preservation. Pooled analyses indicate an OS rate of approximately 56%, a RFS rate around 51%, and a recurrence rate near 15%. Importantly, long‐term renal function is largely maintained—with mean GFR values within normal pediatric ranges—and the need for dialysis or transplantation remains low. However, substantial heterogeneity among study designs, treatment protocols and follow‐up durations underscores the need for standardized, prospective investigations. Until more robust, multicenter data are available, patient selection for nephron‐sparing approaches should be individualized, incorporating tumor response to chemotherapy, anatomical considerations and multidisciplinary expertise to optimize both survival and renal outcomes.

## Author Contributions

Menna Sarhan and Yasmine Adel Mohammed conducted statistical analysis and wrote the main manuscript text. Ayah Abdulgadir and Ibrahim Moqbel designed the research study, defining the main research questions and objectives. Yehia Nabil Abdalla, Khaled Alsayed Abualkhair, and Ahmad Alkheder were responsible for retrieving the relevant literature and selecting studies that met the inclusion criteria and handled data extraction and quality assessment. Salma Allam, Menna Sarhan, and Ahmad Alkheder integrated the results and drafted the discussion section of the manuscript.

## Funding

The authors received no specific funding for this work.

## Disclosure

All authors have read and approved the final version of the manuscript. The corresponding author, Ahmad Alkheder, had full access to all of the data in this study and takes complete responsibility for the integrity of the data and the accuracy of the data analysis.

## Ethics Statement

The authors have nothing to report.

## Consent

The authors have nothing to report.

## Conflicts of Interest

The authors declare no conflicts of interest.

## Transparency Statement

The lead author, Ahmad Alkheder, affirms that this manuscript is an honest, accurate, and transparent account of the study being reported; that no important aspects of the study have been omitted; and that any discrepancies from the study as planned (and, if relevant, registered) have been explained.

## Data Availability

The data that support the findings of this study are available from the corresponding author upon reasonable request.
